# How Well Do Couples Care When They Are Expecting Their First Child? Family and Dyadic Predictors of Parental Sensitivity in Expectant Couples

**DOI:** 10.3389/fpsyt.2020.562707

**Published:** 2020-11-12

**Authors:** Maria Kaźmierczak, Paulina Pawlicka, Ariadna B. Łada-Maśko, Marinus H. van IJzendoorn, Marian J. Bakermans-Kranenburg

**Affiliations:** ^1^Institute of Psychology, Faculty of Social Sciences, University of Gdańsk, Gdańsk, Poland; ^2^Department of Psychology, Education and Child Studies, Erasmus University Rotterdam, Rotterdam, Netherlands; ^3^Clinical Child and Family Studies, Faculty of Behavioural and Movement Sciences, Vrije Universiteit Amsterdam, Amsterdam, Netherlands

**Keywords:** parental sensitivity, family-of-origin, couple, empathy, abuse

## Abstract

Belsky's *Process Model* points to family-of-origin (especially experiences of mistreatment in childhood) as well as personality and marital relations as determinants of parenting quality, including parental sensitive responsiveness. Parental sensitivity might be intuitively developed during pregnancy and affects perinatal mental health. However, there is a lack of studies investigating effects of family-of-origin and relationship perceptions on expectant couples' parental sensitive responsiveness. The aim of the presented study was to test mediation and moderation effects of perceived partner's empathic concern and retrospectively assessed abuse experienced in childhood on sensitive parental responsiveness operationalized as caretaking behaviors and emotional reactions to a crying life-like doll. One hundred eleven expectant couples (*N* = 222; age: *M*_women_ = 28.4 years, *SD* = 3.03; age: *M*_men_ = 29.2 years, *SD* = 3.31; relationship duration: *M*_years_ = 6.8, *SD* = 3.43; gestational week: *M* = 31.3, *SD* = 4.58) assessed the extent to which they experienced physical and emotional abuse from their parents in childhood and rated their current partner's empathic concern. In the experimental procedure, couples reacted to a crying life-like doll and were assessed by trained psychologists using the modified Ainsworth Sensitivity Scale to measure couples' sensitive responsiveness. The results confirmed a significant mediational effect of perceived women's (and not men's) empathic concern for the relationship between the reported experience of abuse in family-of-origin by expectant fathers (and not mothers) and couples' sensitivity. Support and interventions regarding couples' empathy and parenting competence can be offered to both mothers and fathers to identify those who are at risk of low parental sensitivity.

## Introduction

The transition to parenthood is a challenging period for couples. A dyadic relationship transforms into a new triadic family structure where parents individually but also as a couple form bonds with their child. Both individual characteristics of each parent and the quality of their dyadic relationship contribute to this transition experience (1). Expectant parents may already display caregiving behaviors toward their unborn child, reflecting their own representations of their baby and future parental commitment. Such behaviors observed in pregnancy are apparent also in postnatal interactions with a real child (2). Thus, the quality of the triadic family system develops during pregnancy and affects mental health postnatally. Observation of mutual relations between parents-to-be also in the context of parental roles may serve as a valid indicator of their future parental sensitivity and child's attachment (3). Support and interventions regarding parenting competence can be offered to both mothers and fathers already during this prenatal period, before their baby is born.

### Sensitivity to Child's Signals as a Dimension of Parenting Quality

One of the essential dimensions of high-quality parenthood, focusing on close emotional bonds and offering the child a secure base to explore the world, is parental sensitivity to child's signals (4, 5). It refers to the ability to appropriately recognize infants' behavioral and emotional cues and to respond during interaction in a well-timed, reciprocal, and mutually rewarding manner (6, 7). Some studies show that sensitivity toward infant's distress is a better predictor of emotion regulation and secure attachment than sensitivity to non-distress (8), pointing to the importance of parental sensitive responding to a child's crying. In this paper, we test the predictors of couple's sensitivity to infant cues in the dynamic period of transition to parenthood.

### Predictors of Parenting Quality—The Process Model of Parenting

According to Belsky's (9) *Process Model*, parenting quality is predicted by major but not equally powerful sources of influence: *characteristics of the parent* (developmental history including abuse experienced in childhood, personality characteristics), *characteristics of the child*, and the broader *social context of the parent–child relationship* (with a potentially high impact of marital relationship).

#### Partner's Empathic Concern

Originally, the *Process Model of Parenting* emphasized the mediational role of marital relations in the association between parental characteristics and parenting (e.g., parental sensitivity). Here we examine the mediating role of perceived partner's empathic concern as an essential aspect of this social context. Empathic concern was defined by Davis (10) as other-oriented emotional empathy linked with compassion and sympathy for unfortunate others, warmth or being moved by perceiving others in need, also in close relationships characterized by communal sharing (11). Such other-oriented empathy or empathic concern has been a focus of family and developmental research, including research on the quality of intimate relationships [e.g., empathic concern is positively associated with relationship satisfaction and negatively associated with depression among heterosexual couples; (12)]. Empathic concern has also been found to be associated with parenting quality. Higher empathy facilitates concentration on the child's needs (13–15), while lower empathy increases the risk of self-focused behavior (16) and of general disturbances in family relationships including child abuse (17).

In order to take empathic concern into account as part of the social context (social support and relationship quality), we focus on mutual perceptions of partners' empathic concern, not on self-reported empathy. This construct encompasses both positive perception of a partner as supportive and perception of relationship with a partner as satisfying. Busby and Gardner (18) showed that men's perception of their partner's empathy was associated with higher perceived relationship quality in couples in a longitudinal perspective. Additionally, perceived partner's empathic concern is associated with better adaptation of mothers as well as fathers during the transition to parenthood (19). Higher dyadic empathy also correlated with higher sexual satisfaction and relationship adjustment while transitioning to parenthood (20).

#### Caregiving Experiences During Childhood

Both parental sensitive responding and empathic concern are partly dependent on experiences in the family-of-origin (16, 21). Difficulties in fulfilling parental roles, low empathic concern, and low levels of perceived empathic support in the relationship with a romantic partner were linked to negative caregiving experiences or abuse during childhood (sexual, physical, and/or emotional) [e.g., (22, 23, 26)]. For example, mothers who had been maltreated in childhood were more intrusive during mother–child interactions (24), prone to more negative responses, and more frequent abusive behaviors toward their own children (25, 27). Gender might modify the relationship between the family-of-origin and individual outcomes in adulthood (28) or parenting behaviors (29), but it needs to be confirmed in the context of parental sensitivity.

However, the updated *Process Model* suggests additional moderating pathways, as interactions between different predictors (including developmental history of parents and their personality) might impact the quality of parenthood (9, 29). Such moderation is consistent with the *buffering effect model* that points to the beneficial role of social context, which ameliorates the possibly negative impact of stressors on parenting quality (29). Additionally, in earlier studies, effect sizes of parental and child characteristics on parenting were modest to small, whereas the effects of the social context domain (e.g., parents' relationship quality) were stronger, especially in the presence of stressors (29). Therefore, the question remains whether empathic concern may also serve as a buffering factor in the association between negative childhood experiences and parental sensitive responding.

### Present Study

Although many studies have been published on parenthood in the realm of the *Process Model*, the focus on fathers is still insufficient (29, 30), especially in light of the significant impact of an intimate relationship on the paternal role [e.g., (31)]. It should also be noted that Belsky's *Process Model* (9) was based on nonexperimental and correlational research, and there has been an increase in experimental designs in parenting research, from which interventions aimed at improving parenting quality might profit (32). Thus, in the present paper, we focus on negative caregiving experiences during childhood and current partner's perceived empathic concern as predictors of parental sensitivity in expectant couples, measured in a standardized experimental setting.

We hypothesized that negative caregiving experiences during childhood and perceived empathic concern of a partner would predict couple's parental sensitivity during pregnancy (Hypothesis 1). Moreover, according to Belsky's *Process Model*, we predicted that perceived empathic concern of a partner would mediate the relationship between childhood abuse and couple parental sensitivity (Hypothesis 2) or alternatively would serve as a moderator of this association (Hypothesis 3). Depressive symptoms in expectant parents have also been taken into account since the transition to parenthood is closely related with emotional health (33).

## Method

### Participants

A total of 111 young adult couples (*n* = 222) from the Pomeranian region in Poland, who were expecting their first child (week of pregnancy at the time of recruitment: *M* = 31.3, *SD* = 4.58) participated in the study. Women (age: *M*_women_ = 28.40 years, *SD* = 3.03) were significantly younger than men (age: *M*_men_ = 29.24 years, *SD* = 3.31) [*t*_(220)_ = 1.99; *p* = 0.048; Cohen's *d* = 0.26]. The average relationship duration was *M* = 6.8 years (*SD* = 3.43; it ranged from 2 to 16 years). Also, 81% of the participants were married and 19% were in an informal relationship. None of the participants had children from previous relationships. All couples lived together (*M*_years_ = 3.56, *SD* = 2.15; duration ranged from 0.5 to 10.5 years). Also, 83% of the participants had a degree of higher education and 91% of participants were professionally active.

### Procedure

#### Recruitment

The recruitment of couples took place during antenatal classes and through social media. Firstly, all couples willing to participate in the study completed an online recruitment questionnaire, which contained basic sociodemographic information and relationship status. The inclusion criteria were: age range 19 [the beginning of Erikson's early adulthood phase; (34)] until 35 (end of this phase); minimum 2-year relationship duration [as in earlier Polish studies on cohabiting couples; see (35)], sharing a household, and third trimester of pregnancy with a first child. Additionally, participants had to declare general good health and no chronic disease diagnosis, no pharmacological treatment, and no psychoactive substances abuse.

The study took place in the laboratory setting with a two-way mirror and cameras, furnished as a nursery room, situated at the University of Gdansk. After introducing the procedure, all participants signed an informed consent concerning their voluntary participation in the study. Each participant was first requested to assess perceived partner's empathic concern and report negative caregiving experiences during childhood followed by an experimental procedure with an infant simulator. After completion of all tasks, each participant was thanked and received 100 PLN (ca. 25 Euro). The study was approved by the Independent Bioethics Committee for Scientific Research at the Medical University of Gdańsk, Poland (permission # NKBBN/154/2017) and the Ethics Committee at the Institute of Psychology, University of Gdańsk, Poland (permission # 4/2016).

#### Observational Stage

The procedure included a 10-min observation in which a couple was asked to take care of a baby (which was a life-like doll). The infant simulator was programmed to cry with a varying frequency for 7 out of 10 min. The procedure that proved to be perceived as a realistic experience (32, 36) includes a doll with infant features (professional infant simulator) and crying patterns gradually changing from fussing to crying and screaming of varying frequency. Face and criterion validity, convergent and discriminant validity of the procedure has been proven in various low-risk samples (childless undergraduate students, young mothers). The physical presence of the life-like doll facilitated responding of a caregiver as compared to experiments that often used computerized cry sounds (37) as participants talked to the infant simulator or used their name while caregiving. The above standardized procedure allows observation of parents' sensitivity to infant crying (distress signal) in conditions similar to the realistic situation of caring for a baby (32). The entire caretaking procedure was recorded and then coded by trained raters using the *Ainsworth Sensitivity Scale* (38) modified by Voorthuis et al. (32) for coding the sensitivity toward a life-like doll.

### Measures

#### Parental Sensitivity

The 9-point *Ainsworth Sensitivity Scale* (38) in the modified version used by Voorthuis et al. (32) was used for the couple observational assessment of parental sensitivity to an infant simulator's crying. Parental sensitivity was assessed from the perspective of an infant; hence, the rating depended on the sensitivity toward the child's (infant simulator's) needs provided by the couple, regardless of individual ratings of each partner. Couple sensitivity was assessed by a trained rater. Higher scores reflect higher parental sensitivity provided to the life-like doll by a couple. For instance, a score of seven points or higher meant that the baby for majority of the time received a prompt, adequate, and well-rounded care provided by the couple (38). Sensitivity with the infant simulator has been shown to be strongly correlated with sensitivity to a parent's own baby [*r* = 0.53, *p* < 0.01; (36)]. The average intercoder reliability [ICC, two-way random effects, absolute agreement; (39)] for couple sensitivity with the infant simulator in expectant couples was 0.94 (range 0.85–0.97 based on 20% of the sample and 12 coders).

#### Perceived Partner's Empathic Concern

A nine-item measure of perceived empathic concern (40) was used. It was based on the index of empathic concern created by Matthews et al. (41) and used to assess a partner in an intimate relationship. The measure consists of nine adjectives (e.g., helpful, sensitive, sympathetic) with a 5-point Likert scale. The participants were asked to indicate to what extent each of the listed characteristics describes their partner. The higher the score, the higher perceived partner's empathic concern. In this study, Cronbach's α was 0.80.

#### Negative Caregiving Experiences During Childhood

Couples also filled in questions based on the *Short Child Maltreatment Questionnaire* (42) referring to the extent to which they experienced physical and emotional abuse from their mothers and fathers in childhood according to the WHO guidelines (43). The following questions were asked regarding mothers and fathers separately: “*During your childhood, how often did you experience the following behaviors of your mother/father: (1) physical punishment, beatings, jerking, or slapping? (2) insults, placing too high demands, ridiculing*?.” Participants assessed their experience on a 4-point scale (from *never* to *often*). Due to the skewed distribution of variables resulting from the nonclinical population recruitment (declared experience of abuse was rare), results were transformed into two-category variables (experiencing abuse vs. no experience of abuse). Only those participants who declared lack of abuse experience (answered “*never*” to each of the four questions regarding experiences with both mother and father) were assigned to the group with no abuse experience. In this study, Cronbach's α was 0.66.

#### Depressive Symptoms

Depressive symptomatology of both females and males was measured as a control variable. The *Edinburgh Postnatal Depression Scale* [EPDS; Polish validation by (44)], measuring emotional functioning during pregnancy and postnatally [see (45)], was used. This is a 10-item measure with a 4-point response scale. Cronbach's α for the scale was 0.76.

## Results

Table 1 shows the means, standard deviations, and intercorrelations between the study variables. A total of 65 women (58.6%) and 82 men (73.9%) in the study declared having ever experienced maltreatment from their parents during childhood. However, the frequency of maltreatment occurrences was low, and <3% of participants declared *often* experiencing them. No participant in the study reached the threshold score marking perinatal depression. The comparative analyses indicated that women were perceived by their partners as more empathic than men [*t*_(1, 110)_ = −3.03, *p* = 0.003, Cohen's *d* = 0.35] but presented a higher level of depressive symptoms [*t*_(1, 110)_ = 5.83, *p* = 0.000, Cohen's *d* = 0.74], while men reported experiencing abuse during childhood more often [*t*_(1, 110)_ = −2.54, *p* = 0.012, Cohen's *d* = 0.32] than women.

**Table 1:** Means, standard deviations, and intercorrelations between the study variables.

**Variable**	***M***	***SD***	**1**	**2**	**3**	**4**	**5**	**6**
1. Women's experience of abuse	0.59	0.49	–					
2. Men's experience of abuse	0.74	0.44	−0.08^a^	–				
3. Women's empathic concern perceived by her partner	38.17	3.99	−0.07	−0.21^*^	–			
4. Men's empathic concern perceived by his partner	36.55	5.16	−0.07	−0.22^*^	0.26^**^	–		
5. Couple's parental sensitivity	5.48	1.75	0.02	−0.09	0.21^*^	0.14	–	
6. Women's depression	5.07	3.15	0.07	0.02	0.08	−0.10	−0.01	–
7. Men's depression	3.00	2.38	0.02	0.04	−0.19^*^	−0.01	−0.03	0.12

N = 111.

a Phi coefficient.

* p < 0.05,

** *p < 0.01*.

With regard to our first hypothesis, women's (but not men's) empathic concern as perceived by their partners was related to the couple's parental sensitivity during pregnancy. Additionally, for men (but not for women), negative childhood experience correlated negatively with their empathic concern as perceived by the partners and with their ratings of partner's empathic concern. There was no correlation between negative childhood experience and couple's sensitivity for men or for women.

In order to test the hypothesis that perception of partner's empathic concern mediates the relationship between women's and men's reported experience of abuse and couple's parental sensitivity (Hypothesis 2), we used path analysis, controlling for male's and female's depression. Path analysis can be used to analyze models that are more complex (and realistic) than multiple regression. We used the R environment (46) with the lavaan package (47) for calculations.

**Figure 1:**
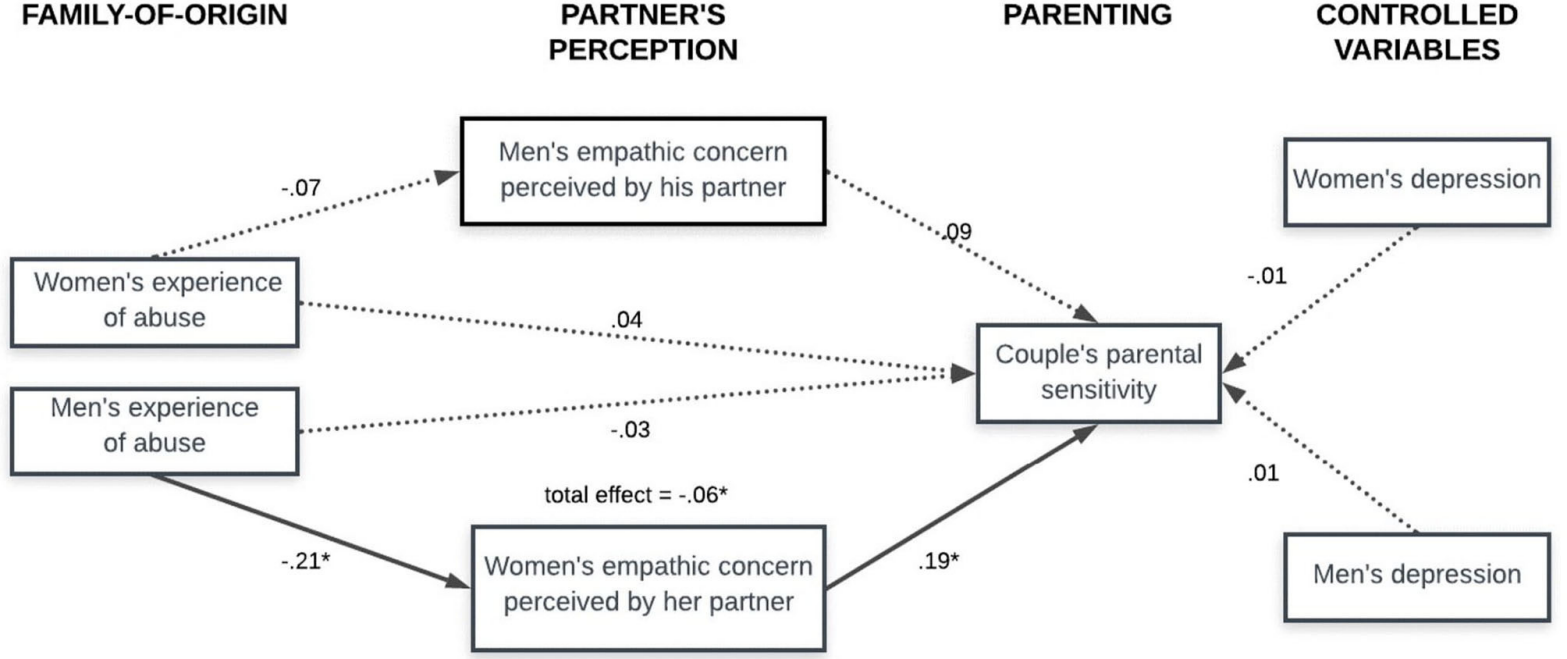
Fully standardized regression coefficients for the relationship between partners' experience of abuse and their parental sensitivity (as a couple) mediated by the perception of the partner's empathic concern. *p < 0.05.

The association between men's reported experience of abuse and couple's parenting sensitivity was mediated by women's empathic concern perceived by their partners. As Figure 1 illustrates, the standardized regression coefficient between men's experience of abuse and women's empathic concern perceived by the men was statistically significant, as was the standardized regression coefficient between women's empathic concern perceived by their partners and couple's parental sensitivity. The standardized indirect effect was (−0.21)^*^(0.19) = −0.04, and the total effect was 0.06. We tested the significance of this indirect effect using bootstrapping procedures. Unstandardized indirect effects were computed for each of 1,000 bootstrapped samples, and the 95% confidence interval was computed by determining the indirect effects at the 2.5th and 97.5th percentiles. The bootstrapped unstandardized indirect effect was −0.16, and the 95% confidence interval ranged from −0.44 to −0.04. Thus, the indirect effect was statistically significant: men with negative childhood experiences perceived their partners as less empathic, and this, in turn, predicted lower levels of couple's parental sensitivity.

As can be seen in Figure 1, the model paths regarding the relationship between women's experience of abuse and couple's parental sensitivity as well as women's experience of abuse and men's empathic concern perceived by the women were not statistically significant. Also, men's empathic concern perceived by their partners was not a significant predictor of couple's parental sensitivity assessed by an independent expert. Since the correlation between mediators was not very high (Table 1), we decided not to include it in the model to simplify it. Also, as can be seen in Figure 1, depressive symptomatology did not significantly add to the prediction of the couple's parenting sensitivity in the model.

To test the hypothesis that women's and men's perceived partners' empathic concern would moderate the association between abuse experienced in childhood and couple's sensitivity to the life-like doll (Hypothesis 3), moderated multiple regression analyses were run. Following the suggestion by Aiken and West (48), predictors were centered, and the interaction term was based on these centered scores. The results of moderated multiple regression analyses showed that there were no interaction effects of women's and men's experience of abuse and perception of partners' empathic concern (B = 0.00, *p* = 0.99 and B = 0.07, *p* = 0.61, respectively).

## Discussion

This is the first study that measured couples' parenting sensitivity during pregnancy, examining what factors contribute to couples' being more or less sensitive to a life-like crying doll.

We found partial support for our first hypothesis. Perceived empathic concern of a partner predicted couple's sensitivity measured experimentally in pregnancy. However, these effects hold only with regard to men, not women. No correlations were found between childhood negative experiences and couple's sensitivity for men or for women.

In addition, our results partially confirmed the mediational pathway converging with Belsky's *Process Model* (Hypothesis 2), where intimate relationship quality, in our case, men's perception of their female partner's empathic concern, acted as a mediator in the association between male retrospectively assessed negative caregiving experiences in childhood and couple's sensitivity toward the crying life-like doll. The mediational pathway referring to woman's childhood experiences, her perception of her partner's empathic concern, and the couple's parenting sensitivity was nonsignificant.

Earlier research showed a significant impact of female empathy or female perceptions of male partner's empathy on romantic relationships quality, also in times of transitioning to parenthood (18, 19, 49), as it was linked with a higher female need for intimacy or with more communal orientations in relationships. We found that when men perceived their partners as more empathic, the couple showed higher levels of parenting sensitivity. Empathy and sensitive responding to a child's needs are often linked with motherhood (50, 51), and in previous research, lack of maternal empathy was related to insensitive parenting reactions to infant crying. Our procedure may have activated the gender role expectations of women taking the responsibility for the crying life-like doll as typical primary carers, whereas men might have tended to rely more on their female partners in the caretaking task. In consequence, females perceived as more empathic might have been easier for men to rely on during the experiment, which led to higher couple sensitivity. Such tendencies might have been enhanced by the fact that the couples were actively preparing for childbirth during antenatal classes, which are typically more woman-centered (52) and might have given a pregnant partner the role of a guide in interacting with “a crying infant.” In Polish culture, motherhood, represented by the stereotype of *a Polish mother*, emphasizes the dominant nurturing role of women (53), while the role of a father is much less stressed. At present, despite growing popularity of egalitarian views on marriage, caring for small children is strongly influenced by the above stereotype (54). The parental role increases after the child's birth for both genders but is more salient for women, while the professional role becomes more salient for men (55).

The mediational pathway might also be interpreted as an indication of a spillover effect between intimate relationship quality and parenting among expectant fathers [e.g., (56)]. In men, romantic satisfaction and received partner support during the transition to parenthood or maternal relational competence have been related to the quality of fatherhood, e.g., the amount of effort invested in paternal role (31, 57). In our case, when an expectant father perceived his partner as more empathic, the caretaking potential of a couple in observational psychological assessment was higher. Finally, it should be noted that couple sensitivity was assessed from the perspective of the infant, which did not take into account gender differences in actual care given by men or women during the observation.

No interactions between recollected childhood experiences and perceived partner's empathic concern were found (Hypothesis 3). Thus, we cannot conclude that perception of partner's empathic concern exerted the buffering effect on the negative influence of childhood abuse on experimentally measured parental sensitivity toward a crying life-like doll. Previous research indicated buffering effects of social support that attenuated the impact of stressors on parenting. However, such results have been obtained mostly in high-risk samples and among mothers (29). Maybe because we studied expectant but still childless couples representing a normative population, the direct effect of any recollections of childhood abuse on caring for a life-like doll in couples was weak. In consequence, the roles of partners and their empathy might have been more pronounced in the context of the experimental task.

Why men's and not women's recollection of negative childhood experiences is associated with perception of partner's empathic concern remains an outstanding issue. Men who reported more negative childhood experiences were seen as less empathic by female partners whom they assessed as less empathic as well. Earlier studies indicated the impact of family-of-origin on intimate relationships in both men and women or stronger effects of childhood on females (58, 59). We might hypothesize that women as *barometers* for distress in marriage (60) might perceive their partners based on particular interactions with them despite their own negative caregiving experiences in childhood. Still, this interpretation remains to be confirmed.

## Limitations and Strengths

The recruitment procedure using antenatal classes might have resulted in higher participation of well-educated and highly involved couples, which might have impacted null findings regarding a direct effect between childhood maltreatment and sensitivity, and could have limited generalizability of the results. Still, examining a non-risk sample provides an opportunity to identify more universal predictors of parental sensitivity. Additionally, the cross-sectional nature of the study did not allow for the conclusions regarding directionality of the mediation model.

The experimental setting with a life-like doll might have been experienced as not realistic for all participants. However, earlier studies confirmed its validity (32, 36), and the procedure enables to control for interfering factors that are typical for real-life interactions between parents and their infant. The present study used observation of triadic relations in a controlled laboratory setting. It also created the unique opportunity to assess parental sensitivity in expectant but still childless couples. This way, the couple's potential for taking care of their child can be assessed before facing this task as parents. To further test our interpretations of the results, inclusion of additional variables (e.g., self-reported relationship satisfaction, individual sensitivity, own parent's accounts regarding parenting styles) would be advisable.

The results of the study point to the importance of couple relations for their sensitive responding to the infant cues. They also highlight the importance of empathic concern displayed and perceived by partners in predicting their parenting quality. Focusing on this aspect of marital functioning in expectant couples creates the opportunity to identify those who are at risk of low parenting sensitivity. It also creates the new and rarely utilized time frame for providing support to enable more mature dealing with challenges of parenthood (61). Therefore, even short empathy sessions for non-risk couples offered during antenatal classes may improve perinatal mental health of a dyad that becomes a triad.

## Data Availability Statement

The raw data supporting the conclusions of this article will be made available by the authors, without undue reservation.

## Ethics Statement

The studies involving human participants were reviewed and approved by the Independent Bioethics Committee for Scientific Research at Medical University of Gdansk, Poland (permission #NKBBN/154/2017) and the Ethics Committee at the Institute of Psychology, University of Gdansk, Poland (permission #4/2016). The patients/participants provided their written informed consent to participate in this study.

## Author Contributions

All authors listed have made a substantial, direct and intellectual contribution to the work, and approved it for publication.

## Conflict of Interest

The authors declare that the research was conducted in the absence of any commercial or financial relationships that could be construed as a potential conflict of interest.
